# Applying an ESSENCE Framework to Understanding Adult Autism Spectrum Disorder and ADHD: Retrospective Parent Reports of Childhood Problems

**DOI:** 10.1155/2013/469594

**Published:** 2013-03-25

**Authors:** Stephanie Plenty, Dag Heurlin, Christina Arlinde, Susanne Bejerot

**Affiliations:** ^1^Department of Clinical Neuroscience, Karolinska Institutet, Stockholm 17177, Sweden; ^2^VUB/KOGNUS, Saint Göran Hospital, Northern Stockholm Psychiatry, Stockholm 11281, Sweden

## Abstract

Diagnoses of autism spectrum disorder (ASD) and attention-deficit/hyperactivity disorder (ADHD) are increasingly being made in adulthood. However, assessments can fail to address the diverse range of problems that patients have experienced. The current study applied an early symptomatic syndromes eliciting neurodevelopmental clinical examinations (ESSENCE) framework to explore retrospectively reported childhood developmental and behavioral problems. It examined if adult ASD and ADHD patients would show problems outside those reflected in the respective diagnostic criteria, and also if these patient groups would show more extensive childhood problems than other psychiatric patients. Parents of adults with ADHD (*n* = 130), ASD (*n* = 57), coexisting ADHD and ASD (*n* = 38), and other psychiatric disorders (*n* = 56) reported on a range of childhood problems. Descriptions of the ADHD, ASD, and ADHD+ASD groups reflected greater impairment than descriptions for patients with other psychiatric disorders in most problem areas. Although differences were observed between ADHD and ASD patients in the core diagnostic areas, these syndromes also shared a number of childhood difficulties. The ESSENCE approach can assist in understanding the symptom history of adult ADHD and ASD patients and can be helpful to distinguish their childhood experiences from other psychiatric patients' experiences.

## 1. Introduction

Adult diagnoses of autism spectrum disorder (ASD) and attention-deficit/hyperactivity disorder (ADHD) have increased in recent decades and are currently estimated to occur in one and four percent of the adult population, respectively [1, 2]. Although these two syndromes are relatively well-known childhood disorders, they have only recently come into the focus of adult psychiatry. Consequently, suitable approaches to understanding the complex needs of these adult patient groups are warranted.

Diagnosis of ASD is based on a qualitative impairment of social interaction and communication as well as restricted repetitive and stereotyped patterns of behavior, interest, and activities [3]. ADHD diagnosis is characterised by attention deficits and/or hyperactivity and impulsivity. Importantly, diagnosis of each of these syndromes requires that the symptoms begin in childhood [3]. Although ADHD and ASD present distinct problems, these two conditions can also appear to share characteristics [4–6], making them sometimes problematic to distinguish. For example, inattention and poor social skills are common to both disorders. However, ADHD and ASD may also share a high rate of comorbidity, with epidemiological studies, for example, estimating 30% prevalence of ADHD amongst ASD patients [7]. 

In addition to symptoms described in the diagnostic criteria, patients with ASD and/or ADHD tend to also experience a range of other behavioral and developmental problems. Both syndromes are associated with language [8, 9] and motor skill deficits [10, 11] as well as mood disorders [12, 13] and sleep problems [14, 15]. They also tend to involve cognitive deficits including executive functions, time perception, and memory functions [16]. Furthermore, ASD and ADHD are thought to have more extensive developmental problems than other psychiatric conditions with typically later onset (such as mood disorders or anxiety disorders) [17, 18].

Both ASD and ADHD are associated with a range of difficulties, of which only some are located within the diagnostic criteria [19, 20]. However, clinical assessments often focus heavily on the core features of a suspected diagnosis and, as a result, can fail to examine the assortment of problems that an individual presents with. Gillberg has conceptualized the variety of problems characterising some young children in clinical settings as ESSENCE (early symptomatic syndromes eliciting neurodevelopmental clinical examinations) [21]. This approach describes the multifaceted developmental and behavioral symptoms often observed in children with neurodevelopmental syndromes, including ASD and ADHD. ESSENCE presents these problems as belonging to eight areas of functioning: (a) general development, (b) communication and language, (c) social interrelatedness, (d) motor skills, (e) attention, (f) activity, (g) behavior (conduct), and (h) mood and/or sleep. Gillberg observes that children presenting for clinical examination with one or more of these difficulties are usually treated by only one type of specialist, although specialists from a range of fields would often be more appropriate [21]. Syndromes such as ASD and ADHD, have significant comorbidities (such as depression) that can be overlooked or misinterpreted in “specialised” assessment and treatment. Consideration of broader problem areas, such as those suggested by ESSENCE, during the diagnostic process of ASD and ADHD would assist in capturing the full picture of an individual's impairments. 

Although ADHD and ASD are usually diagnosed in childhood, adult diagnoses are rapidly increasing [22]. Furthermore, prevalence rates are likely to be higher than current estimates as many adult psychiatric patients go undiagnosed [23]. Consequently, there is a pressing need to extend knowledge in diagnosis and treatment of child ASD and ADHD to adult psychiatry. However, ASD and ADHD symptoms can reduce somewhat [24–26], or can take on new forms with age [27], further complicating assessments. As the diagnoses of ADHD and ASD require that symptoms persist throughout childhood [3], parent reports of childhood symptoms play an important role in the diagnostic process. Therefore, when evaluating adults, it is important that tools are available to assess an adult's broader childhood symptom history. 

This study applies the ESSENCE framework to the adult assessment of ASD and ADHD. In Nordic psychiatry, the five to fifteen (FTF) questionnaire is a widely used instrument that addresses a variety of childhood neurodevelopmental problems [28]. In addition to the inclusion of problems diagnostic of each disorder, the FTF also addresses problem areas that ESSENCE presents as relevant to understanding the full picture of an individual's difficulties [21, 29, 30]. Using the FTF, the current study will explore to what extent retrospective parent reports of childhood symptoms reflect impairments in developmental areas other than those listed in the respective diagnostic criteria. As ESSENCE argues that childhood onset neurodevelopmental disorders such as ASD and ADHD are associated with extensive childhood problems, it is expected that adults with ASD and/or ADHD will have exhibited more childhood problems than patients with other (later onset) psychiatric disorders. The similarities and differences in childhood problems between these diagnostic groups will be examined.

## 2. Materials and Methods

### 2.1. Participants and Procedure

Participants (*n* = 413) were consecutive admissions referred to an outpatient tertiary psychiatric clinic in northern Stockholm (Sweden) by a clinician for diagnosis and treatment of ADHD or ASD. The catchment area of the clinic has a population of nearly 320,000 adult inhabitants from both high and low socioeconomic regions. Self-referrals or patients with an intellectual disability or obvious drug/alcohol problems were not included in the study.

Assessment for adult ADHD and ASD involved clinical interviews and assessments with patients and a parent by certified senior psychiatrists and licensed psychologists, with a diagnosis given after consensus between the two. The DSM-IV diagnostic criteria were applied; however, the criterion limiting concurrent diagnosis of ASD and ADHD was disregarded to allow an investigation of the coexisting ASD and ADHD. The assessment procedure for each patient took 12–18 hours to complete over a 2-week period. Ethics approval was provided by the Regional Ethics Committee in Stockholm.

**Figure 1:**
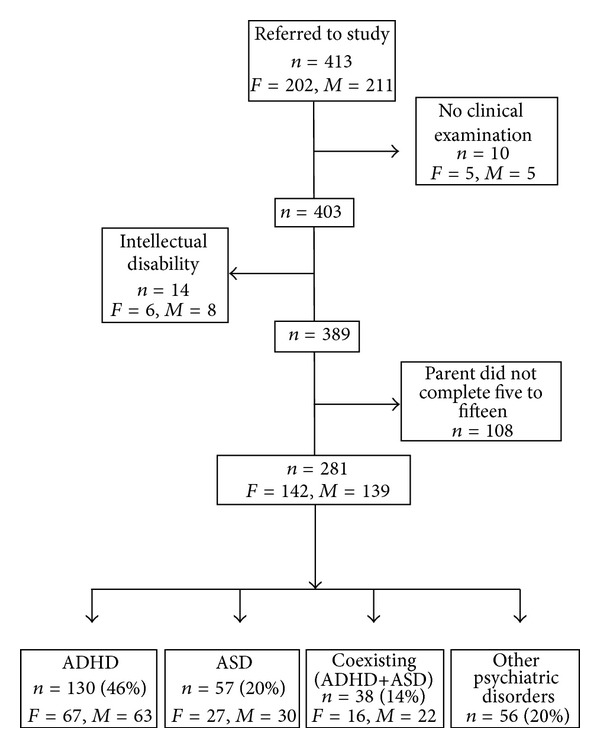
Flow scheme for participation.

At the first consultation, a parent was asked to complete a questionnaire addressing childhood problems (FTF). Parent reports of patients diagnosed by the clinic with ASD (*n* = 57), ADHD (*n* = 130), or coexisting ADHD+ASD (*n* = 38) were compared to a psychiatric disorders group (*n* = 56), mostly comprised of patients with major depression, OCD and other anxiety disorder diagnoses, who were identically assessed but did not receive an ASD and/or ADHD diagnosis. Of the initial sample, 281 participated (females *n* = 142, males *n* = 139; aged between 16 and 57 years of age; mean age = 30.57, SD = 9.86) (see Figure 1).

### 2.2. Diagnosis of ADHD and ASD

In addition to clinical interviews, the following instruments assisted in the diagnosis of ASD and ADHD. For ASD assessment, the Autism Spectrum Screening Questionnaire (ASSQ) [31] and the Asperger Syndrome Diagnostic Interview (ASDI) [32] were completed. The ASSQ is a screening test detecting the high-functioning autism in childhood and is completed by parents [31]. The ASDI is a structured diagnostic patient-interview addressing current symptoms including social impairment, narrow interests, repetitive routines, speech and language peculiarities, nonverbal communication problems, and motor clumsiness [32]. ASDI scores range from 20 to 60. 

For ADHD symptoms, patients completed the Wender Utah Rating Scale (WURS), which addresses childhood symptoms of ADHD [33]. They were also assessed with the Wender-Reimherr Adult Attention Deficit Disorder Scale (WRAADDS) [34]. This structured patient interview addresses adult symptoms including attention difficulties, hyperactivity and/or restlessness, temper, affective lability, emotional overreactivity, disorganization, and impulsivity relating to present difficulties. A summed score (ranging from 0 to 140) is calculated to reflect severity of ADHD symptoms.

In addition, the assessment of general functioning was made using the global assessment of functioning (GAF) [35]. This provides an estimate of the level of social, psychological, and work-related functioning and degree of mental symptoms in the past year. Scores range between 0 and 100, with higher scores representing good functioning in a wider range of activities. The Wechsler adult intelligent scale, neuropsychological version (WAIS III and WAIS III-NI), was used to screen for intellectual disability. 

### 2.3. Childhood Problems: Five to Fifteen Questionnaire

The five to fifteen (FTF) was used to describe a range of developmental and behavioral problems that patients displayed in childhood between ages five and fifteen years [28]. This instrument is a parent-report questionnaire consisting of 181 statements describing neuropsychiatric-neurodevelopmental problems in daily functioning. The FTF has been used extensively in both clinical and research settings, with gender and age norms available [28, 36–38]. Typically, parents complete this questionnaire when their child is being screened for a neurodevelopmental disorder. In the current study, a parent retrospectively completed the FTF about their adult child. Parents were told to focus on when their child was younger than 8 years. This corresponds approximately to the age when symptoms of ADHD or ASD must be present in order to fulfill the diagnostic criteria for these diagnoses and when parents typically recognize these childhood problems. Parents indicated how accurately each statement described their child's behavior in childhood in comparison to what would be considered as normal for this age range. Thus, a behavioral problem noted by the parent when the child was five years but gone at the age of 12 was checked as a problem in the FTF. Reponses are made on a 3-point scale ranging from 0 to 2 (*does not apply, applies sometimes or to some extent, and definitely applies*).

The items represent eight broader domains: (a) motor skills, (b) executive functions, (c) perception, (d) memory, (e) language, (f) learning, (g) social skills, (h), and emotional/behavioral problems. To examine childhood problems in more detail and illustrate the ESSENCE domains, the current study examined 19 FTF subdomains (see Table 2). The subdomain scores were calculated as the mean value of the included subdomain items as described in the FTF questionnaire instructions, with higher scores representing greater childhood difficulties. The current study then compared the median subdomain scores for each group with the FTF median norms available for children aged from 6 to 8 years. For a few subdomains norms for this age group were unavailable as they are related to school performances, and so norms for 9 to 12 years old were used instead (norms available on request at http://www.515.org/). Younger children receive higher ratings than older children [28]; thus, using young age group norms minimized the risk for over-stating the findings in the current study. 

The FTF subdomains of “communication” and “social skills” refer to symptoms in ASD diagnostic criteria, while “attention” and the combined subdomains “hypoactivity and hyperactivity/impulsivity” and “attention” refer to symptoms in ADHD diagnostic criteria.

### 2.4. Statistical Analyses

Statistical analyses were performed in Statistica 7.1 [39]. All significance tests were two-tailed. A series of chi-square tests and Kruskal-Wallis ANOVAs were used to examine patient characteristics between the diagnostic groups. As the scores for each FTF subdomain were highly skewed, non-parametric tests were used. All between-group main effects for each subdomain were analysed using the Kruskal-Wallis ANOVA. Between-group comparisons were analysed pairwise using Mann-Whitney *U* test. A significance *P* value of <.05 was regarded as significant. As the analyses were largely exploratory, the application of a Bonferroni adjustment was not considered as fully applicable in this context.

## 3. Results

### 3.1. Participant Characteristics

The four patient groups shared a similar proportion of males and females as well as age distribution (*P* = .10) (see Table 1). Significantly, more ASD patients were single (*x* ^2^ = 8.35; *df* = 3; *P* = .04) and had low social contact than the other groups (*x* ^2^ = 35.64; *df* = 3; *P* < .001). No significant differences in fulltime employment rates were observed between groups (*P* = .15). Consistent with diagnoses, the ADHD and ADHD+ASD groups showed the highest WRAADDS scores, while the ASD and ADHD+ASD groups showed the highest ASDI scores. Poorer general functioning (lower GAF scores) was observed for the ASD and the ADHD+ASD groups compared to the ADHD group and other psychiatric disorders group (H(3, *n* = 240) = 30.5, *P* < .0001). 

### 3.2. Childhood Problems Reported for ASD and ADHD Patients

To explore the extent to which ASD and ADHD patients were described as having childhood problems beyond those listed in the diagnostic criteria, median subdomain scores for each diagnostic group were compared to the FTF median norms. As shown in Table 2, scores for the ADHD, ASD and ADHD+ASD groups appeared higher than the nonclinical norms on nearly all subdomains. These included the ASD group showing higher “communication” and “social skills” impairment scores and the ADHD group having higher “hypoactivity and hyperactivity/impulsivity” impairment scores than the norms. Three exceptions were observed for “fine motor skills”, “visual perception,” and “time concepts”. 

### 3.3. Childhood Problems in ADHD and ASD Compared with Other Psychiatric Patients

The ADHD, ASD, and ADHD+ASD groups were then compared to patients with other psychiatric disorders to examine if the latter group showed fewer childhood problems. As shown in Table 3, significant main effects were observed on all subdomains. The pairwise comparisons showed that on nearly all subdomains, the ADHD, ASD, and ADHD+ASD groups had higher scores than the other psychiatric disorders group. These included ASD having higher “communication” and “social skills” impairment scores and ADHD having higher “hypoactivity and hyperactivity/impulsivity” and “attention” difficulty scores than the other psychiatric disorders group.

However, there were no significant differences between the ASD group and patients with other psychiatric disorders in “time concepts” and “visual perception”. There were also no differences observed between the ADHD group and the other psychiatric disorders group in “obsessions-compulsions” or “expressive language skills”. Finally, no differences were observed between ADHD+ASD and the other psychiatric disorders group in “obsessions-compulsions”. 

### 3.4. Similarities and Differences between ADHD, ASD, and ADHD+ASD in Childhood Problems

Although the majority of main effects for differences amongst the ASD and ADHD groups were nonsignificant, several differences were observed (see Table 4). Pairwise comparisons showed that the ADHD group showed higher scores than the ASD group in the following five subdomains: “attention” (*P* < .001), “hypoactivity and hyperactivity/impulsivity” (*P* < .01), “planning and organization” (*P* < .001), “time concepts” (*P* < .01), and “memory” (*P* < .05). 

In comparison, the ASD group showed greater difficulties than the ADHD group in “social skills” (*P* < .001). Furthermore, the ADHD+ASD group had higher scores than the ASD group in “attention” (*P* < .01) and “time concepts” (*P* < .05). They also had higher scores than the ADHD group for “communication” (*P* < .05) and “social skills” (*P* < .01).

## 4. Discussion

The current study applied the ESSENCE framework to evaluating the symptom history of adult psychiatric patients assessed for ADHD and ASD. It examined retrospective parent reports of patients' childhood developmental and behavioral problems and found that these descriptions reflected impairments in a range of areas, including and extending beyond those listed in the diagnostic criteria. Furthermore, ASD and ADHD patients showed greater difficulties than patients with other psychiatric disorders in nearly all of the FTF subdomains. In support of ESSENCE, the findings indicate that a range of impairments can be expected in childhood neurodevelopmental disorders such as ADHD and ASD.

The childhood problems described by parents generally reflected impairments characterising the diagnostic criteria for ADHD and ASD. However, the two groups did not differ on “communication” scores. This may be due to the conceptualization of the FTF “communication” items. Only one of the three “communication” items, namely, *“difficulty carrying on a conversation*" describes problems explicitly relevant to ASD, although this can even be problematic for children with ADHD. The other two items refer to abilities to explain events and keep focused when speaking, which are relevant to both ASD and ADHD. ASD however is, characterised by particular difficulties in nonverbal communication (such as eye contact and body language) as well as voice expression, and the FTF addresses these abilities within “social skills”. Although they are theoretically distinct constructs, social skills and communication can be difficult to distinguish in practice and measurement. Consistent with this, the diagnostic criteria for ASD in DSM-5 will merge difficulties in social skills and communication into a single criterion [40].

Our results are consistent with the ESSENCE framework and previous studies showing significant problems for ADHD and ASD patients beyond the diagnostic criteria [6, 41, 42]. Adult patients with ADHD and/or ASD were reported to have had childhood difficulties in motor coordination [10, 11], sleep and externalising/internalising behaviors [12, 13], communication and language (comprehension and expressive problems) as well as in aspects of general development (reading/writing, general learning, and coping in learning) [43, 44]. 

Although the ADHD and ASD groups shared difficulties in several areas of functioning outside the diagnostic criteria, parent reports also revealed trends specific to each condition relating to executive functions. Greater memory function problems were reported for ADHD patients than for ASD patients. This is consistent with impairments reported in other studies for ADHD patients in working memory and long-term memory [42, 45–47] and is likely due to attention and inhibition deficits [46]. Descriptions of ADHD patients also reflected greater difficulty in planning and organizing than those for ASD patients. Although both disorders are associated with problems in these areas [48, 49], the FTF items refer to “perceiving consequences” and “completing tasks”. This focus incorporates impulsivity and possibly explains why ADHD patients' scores reflected greater impairment. A third area of difference was in “time concept”, which involves skills in concentration, inhibition, and memory [50, 51], and is important for orientating to future goals and working towards them [52]. Although difficulties in relating to time are described for both ASD and ADHD [50, 51], in the current study, they were more relevant to adult ADHD and ADHD+ASD patients. 

The current findings illustrate that ASD and ADHD share many problems during childhood that may often be overlooked in specialised treatment and research focusing on exclusively one of these two specific disorders. However, there are some limitations that should be considered when evaluating the findings. Firstly, participants in this study were diagnosed in adulthood. As individuals diagnosed earlier in development (childhood or adolescence) tend to have a more severe condition than those diagnosed at a later stage [53], our participants may have relatively limited impairments compared to the broader ADHD and ASD population. However, they can be considered representative of the Swedish adult population seeking assessment for ADHD and ASD.

Secondly, there is a possibility that parent reports may be influenced by their adult child's symptoms rather than purely reflecting childhood difficulties. Given that all differences amongst the diagnostic groups were in the expected direction (although symptoms for both ADHD and ASD can decrease with age), this influence appears unlikely. Future research could further validate the retrospective use of the FTF by using a prospective design comparing parent reports made in childhood and adulthood.

Thirdly, as multiple comparisons were made, there was an increased risk of making a type-I error, falsely rejecting the null hypothesis. However, given that the analyses were largely exploratory, the application of a Bonferroni adjustment was not considered as fully applicable. Nevertheless, if such an adjustment had been applied, nearly all of the current findings would still apply.

## 5. Conclusion

The current study adds to the evidence that neurodevelopmental syndromes such as ADHD and ASD are associated with a considerable range of developmental and behavioral problems. It also extends prior research by demonstrating that adult ADHD and ASD patients are likely to have displayed more difficulties in childhood when compared to other psychiatric patients and that retrospective parent reports can assist in building a symptom history. The ESSENCE framework and instruments such as the FTF appear to be useful for identifying areas of difficulty that are not diagnostic of ADHD and/or ASD but are nevertheless important for understanding the complexity of patients' needs. 

## Figures and Tables

**Table 1:** Participant characteristics for each patient group.

Patient characteristics	ADHD (*n* = 130)	ASD (*n* = 57)	ADHD+ASD (*n* = 38)	Other psychiatric disorders (*n* = 56)
Gender—male	63 (48.5)	30 (52.6)	22 (57.9)	24 (42.9)
Mean age (SD)	30.2 (10.2)	29.8 (9.8)	29.7 (9.5)	32.9 (9.1)
Single marital status	99 (79.8)	51 (94.4)	27 (75.0)	37 (75.5)
Work full-time	21 (17.8)	5 (9.8)	3 (9.1)	11 (23.9)
Meet friends less than monthly	12 (10.9)	27 (54.0)	12 (37.5)	10 (23.8)
Current symptoms and functioning				
WRAADDS	78.79 (23.74)	46.88 (19.52)	72.84 (24.81)	48.53 (22.04)
ASDI	25.32 (5.09)	37.75 (6.73)	33.05 (7.53)	25.05 (4.24)
GAF	55.53 (10.97)	45.42 (10.87)	47.44 (10.97)	54.27 (12.62)

Characteristic results = *n* (%); current symptoms and functioning = mean (SD).

**Table 2:** Five to fifteen subdomain median scores.

ESSENCE domain	FTF subdomain	ADHD	ASD	ADHD+ASD	Other psychiatric disorders	FTF norms
Motor coordination	Gross motor skills	0.29	0.43	0.50	0.07	.14
Motor coordination	Fine motor skills	0.20	0.10	0.35	0.00	.10
Attention	Attention	1.2	0.60	1.22	0.22	.25
Activity	Hypoactive and hyperactive/impulsive	0.69	0.46	0.50	0.15	.00 and .22*
General development	Planning and organizing	1.17	0.67	1.00	0.00	.33
Motor coordination	Relation in space	0.20	0.20	0.60	0.00	.00
General development	Time concepts	0.50	0.00	0.50	0.00	.05
Motor coordination	Body perception	0.40	0.40	0.40	0.00	.00
General development	Visual perception	0.00	0.00	0.00	0.00	.00
General development	Memory	0.55	0.27	0.45	0.09	.18
Communication and language	Comprehension	0.20	0.20	0.40	0.00	.00
Communication and language	Expressive language skills	0.08	0.08	0.15	0.00	.00
Social interaction	Communication	0.33	0.33	0.83	0.00	.00
General development	Reading/writing and math	0.62	0.23	0.69	0.00	−.12 and −.00*
General development	General learning	0.50	0.50	0.75	0.00	−.00
General development	Coping in learning	1.00	0.70	1.00	0.20	−.20
Social interaction	Social skills	0.44	0.74	0.70	0.15	.04
Behavior/sleep/mood-mood	Internalising and externalising	0.58	0.48	0.44	0.20	.00 and .08*
Behavior/sleep/mood	Obsessive-compulsive	0.13	0.25	0.13	0.00	.00

*FTF norms for 9–12 years old because 6–8 years old norms are not available for these subdomains.

**Table 3:** Comparisons in childhood problems between ADHD/ASD groups and other psychiatric disorders group.

FTF subdomains	Main Effects	ADHD	ASD	ADHD+ASD
	*P *	*P *	*P *	*P *
Gross motor skills	<0.001	<0.01	<0.001	<0.001
Fine motor skills	<0.001	<0.001	<0.05	<0.001
Attention	<0.001	<0.001	<0.001	<0.001
Hypoactive and hyperactive/impulsive	<0.001	<0.001	<0.001	<0.001
Planning and organizing	<0.001	<0.001	<0.01	<0.001
Relation in space	<0.001	<0.001	<0.05	<0.001
Time concepts	<0.001	<0.001	n.s.	<0.001
Body perception	<0.001	<0.001	<0.001	<0.001
Visual perception	<0.01	<0.01	n.s.	<0.01
Memory	<0.001	<0.001	<0.001	<0.001
Comprehension	<0.001	<0.001	<0.01	<0.001
Expressive language skills	<0.05	n.s.	<0.05	<0.01
Communication	<0.001	0.01	<0.001	<0.001
Reading/writing and math	<0.001	<0.001	<0.05	<0.001
General learning	<0.001	<0.001	<0.001	<0.001
Coping in learning	<0.001	<0.001	<0.001	<0.001
Social skills	<0.001	<0.001	<0.001	<0.001
Internalising and externalising	<0.001	<0.001	<0.001	<0.001
Obsessive-compulsive	<0.05	n.s.	<0.01	n.s.

Comparisons between the other psychiatric disorders and the ADHD, ASD, and ADHD+ASD groups; n.s.: nonsignificant; overall group differences were calculated using the Kruskal-Wallis ANOVA; pairwise group comparisons were made using the Mann-Whitney test.

**Table 4:** Main effects for comparisons amongst ADHD and ASD groups.

FTF subdomain	Main effects
	*P *
Gross motor skills	n.s
Fine motor skills	n.s
Attention	<0.001
Hypoactive and hyperactive/impulsive	0.006
Planning and organizing	0.002
Relation in space	n.s
Time concept	0.007
Body perception	n.s
Visual perception	n.s
Memory	0.048
Comprehension	n.s
Expressive language skills	n.s
Communication	0.033
Reading/writing and math	n.s
General learning	n.s
Coping in learning	n.s
Social skills	<0.001
Internalising and externalising problems^r^	n.s
Obsessive-compulsive	n.s

Comparisons amongst ADHD, ASD, and ADHD+ASD groups; overall group differences were calculated using the Kruskal-Wallis ANOVA; pairwise group comparisons were made using the Mann-Whitney test and presented in text.
